# Targeted mass spectrometry for monitoring of neural differentiation

**DOI:** 10.1242/bio.058727

**Published:** 2021-08-06

**Authors:** Rita Sucha, Martina Kubickova, Jakub Cervenka, Marian Hruska-Plochan, Dasa Bohaciakova, Katerina Vodickova Kepkova, Tereza Novakova, Katerina Budkova, Andrej Susor, Martin Marsala, Jan Motlik, Hana Kovarova, Petr Vodicka

**Affiliations:** 1 Laboratory of Applied Proteome Analyses and Research Center PIGMOD, Institute of Animal Physiology and Genetics of The Czech Academy of Sciences, Rumburska 89, Libechov CZ-27721, Czech Republic; 2 Department of Cell Biology, Faculty of Science, Charles University, Albertov 6, Prague CZ-12843, Czech Republic; 3 Department of Quantitative Biomedicine, University of Zurich, Winterthurerstrasse 190, Zürich CH-8057, Switzerland; 4 Department of Histology and Embryology, Faculty of Medicine, Masaryk University, Kamenice 753/5, Brno CZ-62500, Czech Republic; 5 Laboratory of Biochemistry and Molecular Biology of Germ Cells, Institute of Animal Physiology and Genetics of The Czech Academy of Sciences, Rumburska 89, Libechov CZ-27721, Czech Republic; 6 Neuroregeneration Laboratory, Sanford Consortium for Regenerative Medicine, Department of Anesthesiology, University of California, San Diego, 2880 Torrey Pines Scenic Dr., La Jolla, CA 92037, USA; 7 Laboratory of Cell Regeneration and Plasticity and Research Center PIGMOD, Institute of Animal Physiology and Genetics of The Czech Academy of Sciences, Rumburska 89, Libechov CZ-27721, Czech Republic

**Keywords:** Neural stem cell, Neural differentiation, Selected reaction monitoring, Mass spectrometry, Cell line characterization, Protein marker

## Abstract

Human multipotent neural stem cells could effectively be used for the treatment of a variety of neurological disorders. However, a defining signature of neural stem cell lines that would be expandable, non-tumorigenic, and differentiate into desirable neuronal/glial phenotype after *in vivo* grafting is not yet defined. Employing a mass spectrometry approach, based on selected reaction monitoring, we tested a panel of well-described culture conditions, and measured levels of protein markers routinely used to probe neural differentiation, i.e. POU5F1 (OCT4), SOX2, NES, DCX, TUBB3, MAP2, S100B, GFAP, GALC, and OLIG1. Our multiplexed assay enabled us to simultaneously identify the presence of pluripotent, multipotent, and lineage-committed neural cells, thus representing a powerful tool to optimize novel and highly specific propagation and differentiation protocols. The multiplexing capacity of this method permits the addition of other newly identified cell type-specific markers to further increase the specificity and quantitative accuracy in detecting targeted cell populations. Such an expandable assay may gain the advantage over traditional antibody-based assays, and represents a method of choice for quality control of neural stem cell lines intended for clinical use.

## Abstract (precis)

**Summary:** We developed an adaptable mass spectrometry-based assay to assess the differentiation potential of human neural stem cells.

## INTRODUCTION

Neurological disorders affect approximately one-sixth of the human population (United Nations. Nearly 1 in 6 of world's population suffer from neurological disorders – UN report, 2007), and represent a major economic burden for society (United Nations. Nearly 1 in 6 of world's population suffer from neurological disorders – UN report, 2007; Wittchen et al., 2011; World Health Organization. Neurological disorders: public health challenges, 2006). Since the figures are expected to grow (World Health Organization. Neurological disorders: public health challenges, 2006), it is of utmost importance to develop an effective therapy, as currently this is mostly limited to symptomatic treatment, physiotherapy, and occasional surgical interventions. The adult central nervous system (CNS) was long considered a relatively static tissue with very limited regenerative capacity. Nevertheless, ground-breaking discoveries throughout the past two decades demonstrated that in humans, new neurons were produced continuously from neural stem cells (NSCs) residing mainly in the subventricular zone, in the dentate gyrus of the hippocampus (Doetsch et al., 1999; Eriksson et al., 1998; Johansson et al., 1999), and possibly in the striatum (Ernst et al., 2014). Human NSCs can be derived from the fetal CNS, embryonic stem cells (ESCs), or induced pluripotent stem cells (iPSCs), and such *in vitro*-propagated cells survive, divide, migrate, and differentiate into neurons and glial cells in host CNS tissues upon transplantation (Carpenter et al., 1999; Flax et al., 1998; Kobayashi et al., 2012; Svendsen et al., 1997; Vescovi et al., 1999; Yuan et al., 2013; Zhang et al., 2001).

*In vitro*-propagated NSCs cultured in monolayer require fibroblast growth factor-2 (FGF-2) and/or epidermal growth factor (EGF) to survive, retain multipotentiality, and neurogenic efficiency (Carpenter et al., 1999; Conti and Cattaneo, 2010; Flax et al., 1998; Vescovi et al., 1999). Simple withdrawal of the mitogens leads to a spontaneous differentiation mainly into neurons, then astrocytes, and oligodendrocytes (Cattaneo and McKay, 1991; Vescovi et al., 1999; Zhang et al., 2001). Differentiated cells die in the absence of FGF-2 (Vescovi et al., 1999), which can be prevented by using either low levels of FGF-2 (Vescovi et al., 1999) or supplements such as N-2 or serum (Carpenter et al., 1999; Flax et al., 1998), trophic factors such as brain-derived neurotrophic factor (BDNF), glial cell-derived neurotrophic factor (GDNF), nerve growth factor or signalling molecules such as dibutyryl cyclic AMP (Lee et al., 2007; Yuan et al., 2011). Other protocols were developed to direct the NSC differentiation towards particular neural cell types, such as using fetal bovine serum (FBS) together with the N-2 supplement for astrocytes (Meyer et al., 2014). NSCs can also be ‘primed’ or ‘pre-differentiated’ to enrich for cells of particular interest (Yuan et al., 2011), or genetically modified to overexpress relevant proteins (Klein et al., 2005), and this self-production and/or secretion of protein(s) may significantly affect the uniformity of such cell lines.

NSCs can be derived from multiple sources, and properties of such NSC lines differ (Conti and Cattaneo, 2010). Many protocols generate a rather heterogeneous population containing NSCs, committed neuronal and glial cells, or neural crest cells. In the case of ESC- or iPSC-derived NSCs, residual undifferentiated pluripotent stem cells can also be present in cultures, which may cause tumour formation after *in vivo* transplantation (Yabut and Pleasure, 2016). Thus, both differentiation potential and purity of human NSC lines should be periodically screened during the production period, and only a population of NSCs that fulfils the release criteria used for *in vivo* grafting assays.

To develop a potent, specific, and predictable screening assay that defines the NSCs clones of high purity, several criteria need to be met, including the ability to (i) define the NSCs population by the presence of specific markers, (ii) identify the presence of pluripotent stem cells or other cell type contaminants, including the mesoderm and endoderm derivatives, and (iii) offer a quick turnaround from data analysis to interpretation.

Morphology of live cells in culture is regularly checked as a part of good laboratory practice. Next-generation (deep) RNA sequencing offers the potential for a detailed characterization of human NSC lines and for the discovery of novel NSC markers (Bohaciakova et al., 2019). Deep RNA sequencing, however, is currently not fast enough to serve as a screening method, and protein effector levels can be predicted from the RNA levels only with limited accuracy. Although traditional antibody-based screenings such as immunofluorescence (IF) imaging, western blotting, or microarrays are well established for the detection of proteins, their throughput potential is relatively low. Immunoassays such as ELISA or flow cytometry may increase the throughput, but their multiplexing capacity is limited (Kupcova Skalnikova et al., 2017). Mass cytometry, flow cytometry augmented by mass spectrometry (MS)-based detection improves multiplexing potential. Imaging mass cytometry, a technique combining IF and mass cytometry (Bodenmiller, 2016), allows for simultaneous and spatially-resolved quantification, but cannot ensure rapid read-out and analysis.

The application of quantitative proteomics provided essential insights into NSC biology, generating a number of differential protein maps and partial functional networks (Shoemaker and Kornblum, 2016; Zizkova et al., 2015). MS-based quantifications following enrichment strategies for capturing candidate markers of NSCs were performed (Melo-Braga et al., 2014; Song et al., 2019; Tyleckova et al., 2016) using a conventional shotgun approach, where a subset of peptides was automatically and in part stochastically measured in the process of data-dependent precursor selection (Aebersold and Mann, 2003). Recently, we applied the data-independent acquisition MS method that combined global feature detection with targeted data extraction to simultaneously quantify thousands of proteins in the course of NSC differentiation (Červenka et al., 2021). This altogether helped to improve our understanding of the NSC differentiation and to identify potential protein markers of distinct steps in this process. However, such studies are not suitable for routine cell line characterization due to time requirements for data processing.

We aimed to develop an assay that would allow fast, efficient, and accurate monitoring of human NSC cultures using a targeted MS approach based on selected reaction monitoring (SRM). The essence of the SRM is the generation of specific, quantitative MS assays for each protein of interest and their subsequent application to multiple samples (Lange et al., 2008). To achieve this, several independent proteotypic (detectable and unique) peptides of the same protein are targeted, substantially increasing the confidence in the specific detection. The endogenous peptides are measured together with isotopically labelled reference peptides, and their quality can be verified by a fragment ion spectrum. Multiple data points are integrated to quantify proteins of interest, increasing the method statistical power and the precision of determined abundance changes. All this offers higher data reliability compared to the antibody-based methods routinely used for protein quantification. Samples can be processed in a single 30-min multiplexed MS method which makes it possible to collect and analyse the data about a cellular state in a matter of hours without the computational overhead (Soste et al., 2014).

Here we present a novel SRM assay to measure qualitatively and quantitatively the levels of protein markers broadly used to probe neural differentiation, i.e. POU domain, class 5, transcription factor 1 (POU5F1; also known as octamer-binding transcription factor 4, OCT4), transcription factor SOX-2 (SOX2), nestin (NES), doublecortin (DCX), tubulin beta-3 chain (TUBB3), microtubule-associated protein 2 (MAP2), protein S100-B (S100B), glial fibrillary acidic protein (GFAP), galactocerebrosidase (GALC), and oligodendrocyte transcription factor 1 (OLIG1). Such assay can be used to monitor the purity and the differentiation potential of human NSCs, and to identify their optimal culture conditions.

## RESULTS

### Markers selection and SRM method development

We aimed to target a set of protein markers routinely used in NSC differentiation studies (Table S1), including ESC markers (homeobox protein NANOG, NANOG; OCT4), NSC markers (SOX2; NES; paired box protein Pax-6, PAX6; proliferation marker protein Ki-67, MKI67), neuronal markers (DCX, TUBB3, MAP2), astrocyte markers (GFAP, S100B), and oligodendrocyte markers (GALC, OLIG1). We also intended to test the ability to detect low-abundant proteins previously found in our differentiation experiments, namely vascular endothelial growth factor A (VEGF-A) (Červenka et al., 2021) and growth-regulated alpha protein (CXCL1) (unpublished work, Institute of Animal Physiology and Genetics of The Czech Academy of Sciences).

The level of endogenous peptides is typically stoichiometric to the level of proteins (quantotypic). We developed SRM assays using heavy-labelled synthetic reference peptides (Table S2) that do not recapitulate the complexity of post-translational or translational modifications. Incomplete digestion during sample processing may also impact the quantotypic properties, so we performed preliminary measurements of NSCs differentiated with BDNF and GDNF for 21 days. This allowed us to spot discrepancies, exclude outlier peptides (if present), and ensure accurate quantification of protein levels.

**Fig. 1.**
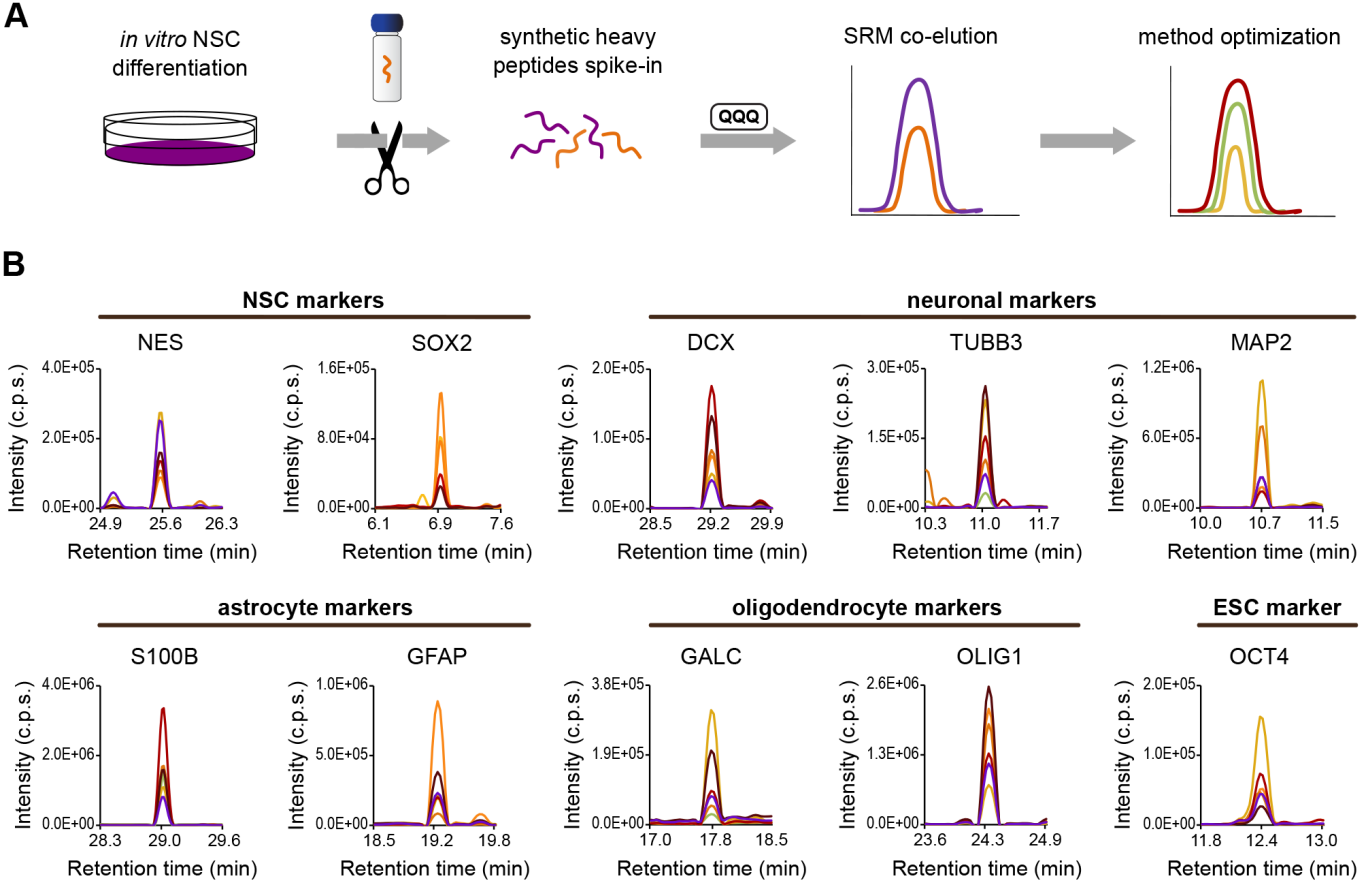
**Design of multiplexed SRM method.** (A) Synthesized peptides (orange string in the vial) containing a heavy-isotope label were spiked into peptide mixtures extracted from differentiating NSCs after trypsin cleavage (purple strings). These samples were measured by SRM on a triple quadrupole to monitor the chromatographic co-elution of endogenous peptides (purple peak) and spiked-in heavy surrogates (orange peak), and a match in relative intensities of fragment ions. Multiple coloured traces in the method optimization graph represent the detection of different fragment ions from common peptide precursor (SRM transitions). (B) Optimal coordinates were assembled into a multiplexed method, and representative heavy peptides of protein markers are displayed.

**Fig. 2.**
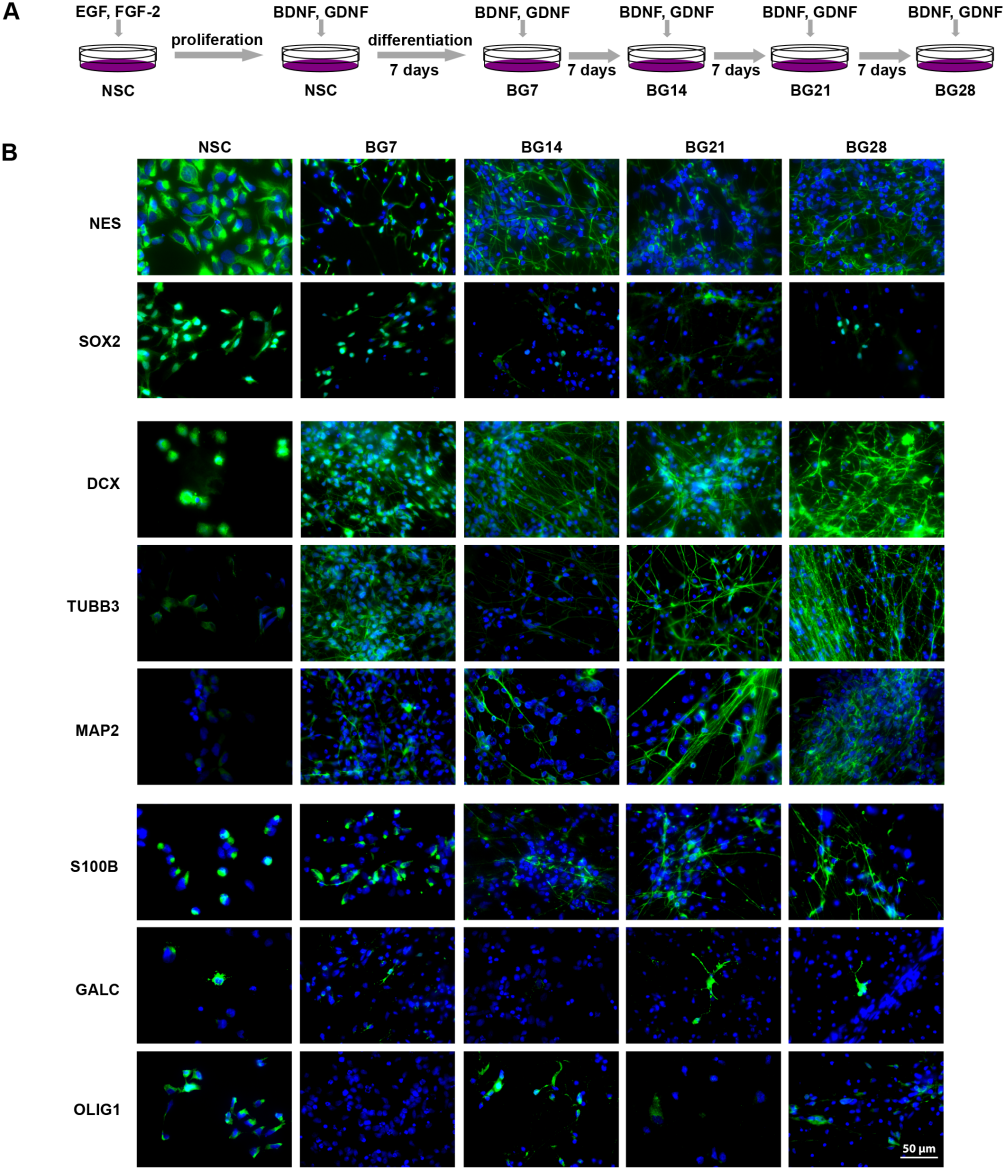
**NSC differentiation analysed by immunocytochemistry.** (A) Scheme of NSC differentiation into neurons by the exchange of EGF and FGF-2 for BDNF and GDNF (BG) for 7, 14, 21, and 28 days. (B) Representative IF images of BG differentiation show protein markers in green; cell nuclei counterstained by DAPI in blue. Scale bar: 50 μm. Images of negative controls (no primary antibody) are shown in Table S3 with the table of used antibodies.

**Fig. 3.**
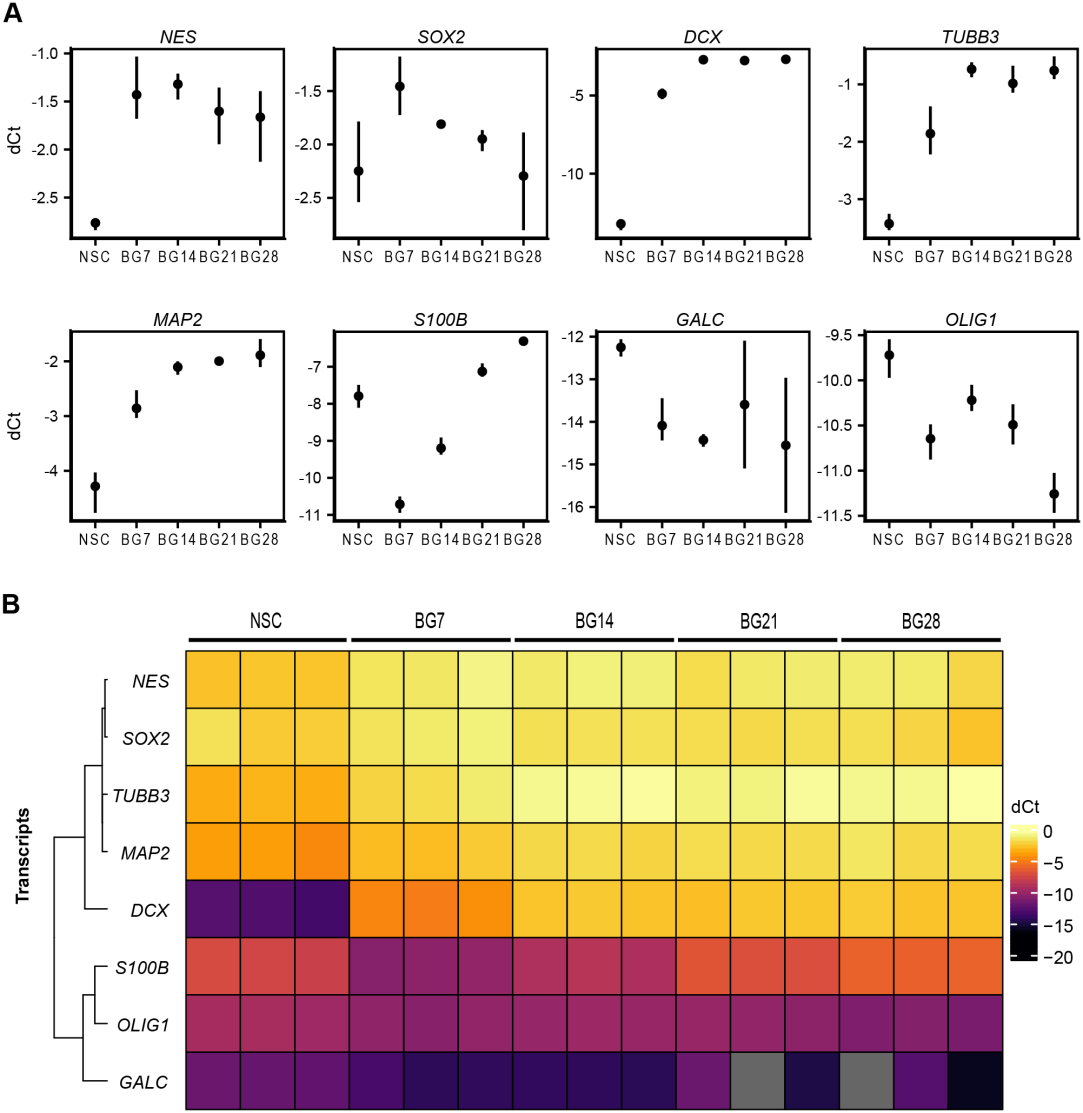
**Analysis of gene expression during NSC differentiation.** (A) mRNA levels analysed by RT-qPCR. Individual transcripts were normalized to two housekeeping mRNA controls (*GAPDH* and *ATP5F1B*). Data from three independent experiments are displayed as mean (point) dCt values ±95% confidence intervals (vertical lines). (B) A heatmap of normalized dCt values from A shows similar co-expression profiles of neural and glial markers over the course of *in vitro* differentiation. Primers are listed in Table S4.

For quantitative measurements, we had selected proteins successfully detected by SRM in our conditions (Fig. 1), and evaluated their capability to provide a read-out for NSCs and their differentiated counterparts by immunocytochemistry (Fig. 2; Table S3), and by gene expression analysis (Fig. 3; Table S4). Then, we assembled optimal coordinates of specific assays for ten markers (OCT4, SOX2, NES, DCX, TUBB3, MAP2, GFAP, S100B, GALC, OLIG1) into a multiplexed SRM method (Table S5). Proteins were represented by two to eight proteotypic peptides with good quantotypic properties, accurately representing the abundance level, and their four to ten most suitable transitions.

### BDNF and GDNF differentiation defined by immunocytochemistry and gene expression analysis

NSCs generated from the NIH approved human ESCs line H9 were cultured in the NSC proliferation medium with EGF and FGF-2. The cells were directed into neurons using the differentiation medium without EGF and FGF-2, and supplemented by BDNF and GDNF (BG) to support cell survival for 7, 14, 21, and 28 days (Fig. 2A). To evaluate the cellular identity of proliferating NSCs and differentiating cells at protein and transcript levels, we applied antibody-based IF imaging and quantitative reverse transcription polymerase chain reaction (RT-qPCR).

All protein markers analysed, excluding OCT4 and GFAP, were detected in NSCs by IF imaging, fibrillar localization of NES, DCX, TUBB3, MAP2, and S100B was mainly apparent in differentiating cells, and only sporadic positivity for GALC and OLIG1 was detected in the later stages (Fig. 2B). Once the BG differentiation had been triggered, the mRNA level of neuronal (DCX, TUBB3, and MAP2) and NSC (NES, SOX2) markers was strongly induced (Fig. 3A). In the second week, the expression of neuronal markers had further increased, and remained stably high, while the expression of NSC markers had gradually decreased (Fig. 3A). Glial markers had dropped in the first week which was followed by steeply rising levels of the astrocyte marker S100B but steady levels of oligodendrocyte markers (GALC, OLIG1) (Fig. 3A).

The clustering of expression profiles (Fig. 3B) showed a separation of NSC (NES, SOX2) and neuronal (DCX, TUBB3, MAP2) markers from glial lineage markers (S100B, OLIG1, GALC). As we expected, our IF and RT-qPCR data showed an induced expression of neuronal markers and a reduced expression of glial markers at the early stages of neuronal differentiation, which was followed by a reduced expression of NSC markers in the later stages.

### BDNF and GDNF differentiation defined by SRM

**Fig. 4.**
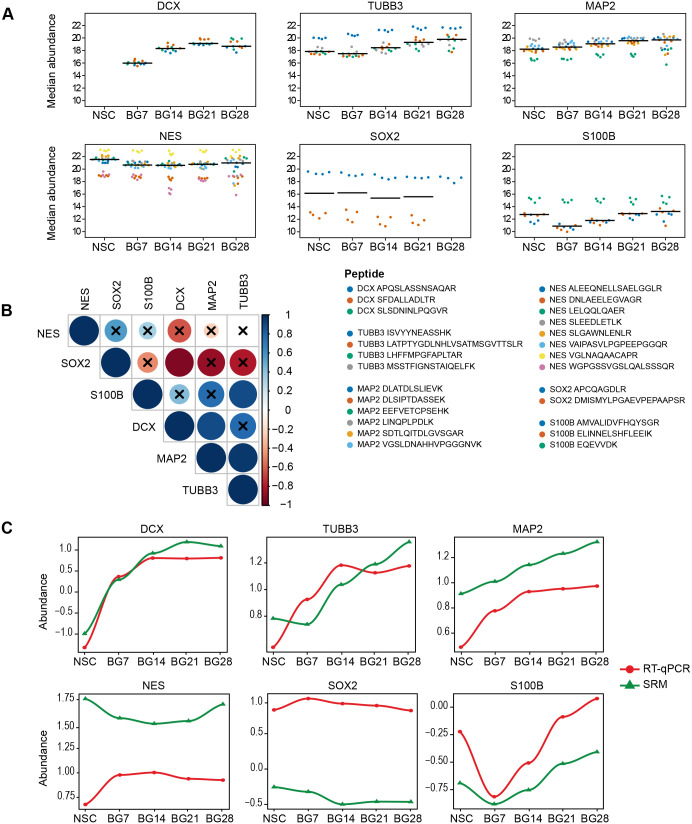
**Simultaneous quantification of 28-day BG differentiation.** (A) Median abundances (black lines) of each protein marker in a given time interval of BG differentiation (7, 14, 21, 28 days). Dots of the same colour represent peptide abundances in four biological replicates. This is defined as the median of log2-transformed peak area of all transitions of the same peptide. Quantification results can be found in Table S6. (B) A correlogram is depicting pair-wise Pearson correlations of individual protein markers over the differentiation time-course. Colour and dot sizes indicate correlation strength, correlations without cross are statistically significant (*P*<0.05). (C) Plots are depicting a correlation of the transcripts levels (RT-qPCR, data from Fig. 3A) and the proteins levels (SRM, data from Fig. 4A) over time. The dCT values for mRNA levels and the log2-transformed values (abundances) for proteins were scaled and centred to mean 0 and standard deviation 1 across all measured targets to allow display in the same graph. A table of corresponding Pearson correlation coefficients is provided as Table S7.

The BG differentiation peptide samples were subjected to simultaneous quantitative measurement by SRM (Table S6). Only proteins detected with ≥2 peptides in either BG differentiated cells or control cells (NSCs) were assigned as quantifiable. This included neuronal and NSC markers (DCX, TUBB3, MAP2, NES, SOX2), and the astrocyte marker S100B (Fig. 4A). If only one peptide of a protein had been detected, this marker was assigned as detectable in a particular condition (GFAP, GALC, OLIG1) (Table S6). In agreement with IF imaging results, OCT4 was not detected by SRM in BG differentiating NSCs (Table S6).

DCX was quantifiable only in differentiating BG cells and not in proliferating NSCs, reaching its maximum level after 3 weeks of differentiation with the highest abundance change recorded in our study. MAP2 and TUBB3 were gradually rising from day 7 and 14, respectively. NES and SOX2 were decreasing from day 7 and 14, respectively. Only one of two analysed SOX2 peptides remained detectable after 4 weeks of differentiation. SRM quantification results show that the method enables monitoring of NSC differentiation (Fig. 4A). All neuronal markers are increased in differentiating BG cells, and all NSC markers are decreased in these cells.

A significant positive correlation over the differentiation time-course was observed for the neuronal markers TUBB3 and MAP2, DCX and MAP2, but also for the glial marker S100B with TUBB3 (Fig. 4B). Despite differences in the S100B peptides performance (Fig. 4A), the changes at the protein level reliably reflected the changes at the mRNA level (Fig. 4C). The S100B protein level decreased in the first week, and returned to its original level in the later stages of BG differentiation (Fig. 4A). The levels of DCX and MAP2 measured by SRM also positively correlated with mRNA levels measured by RT-qPCR (Fig. 4C; Table S7). The significant negative correlation of SOX2 versus DCX (Fig. 4B) confirms the switch from NSCs to differentiating neuronal states.

**Fig. 5.**
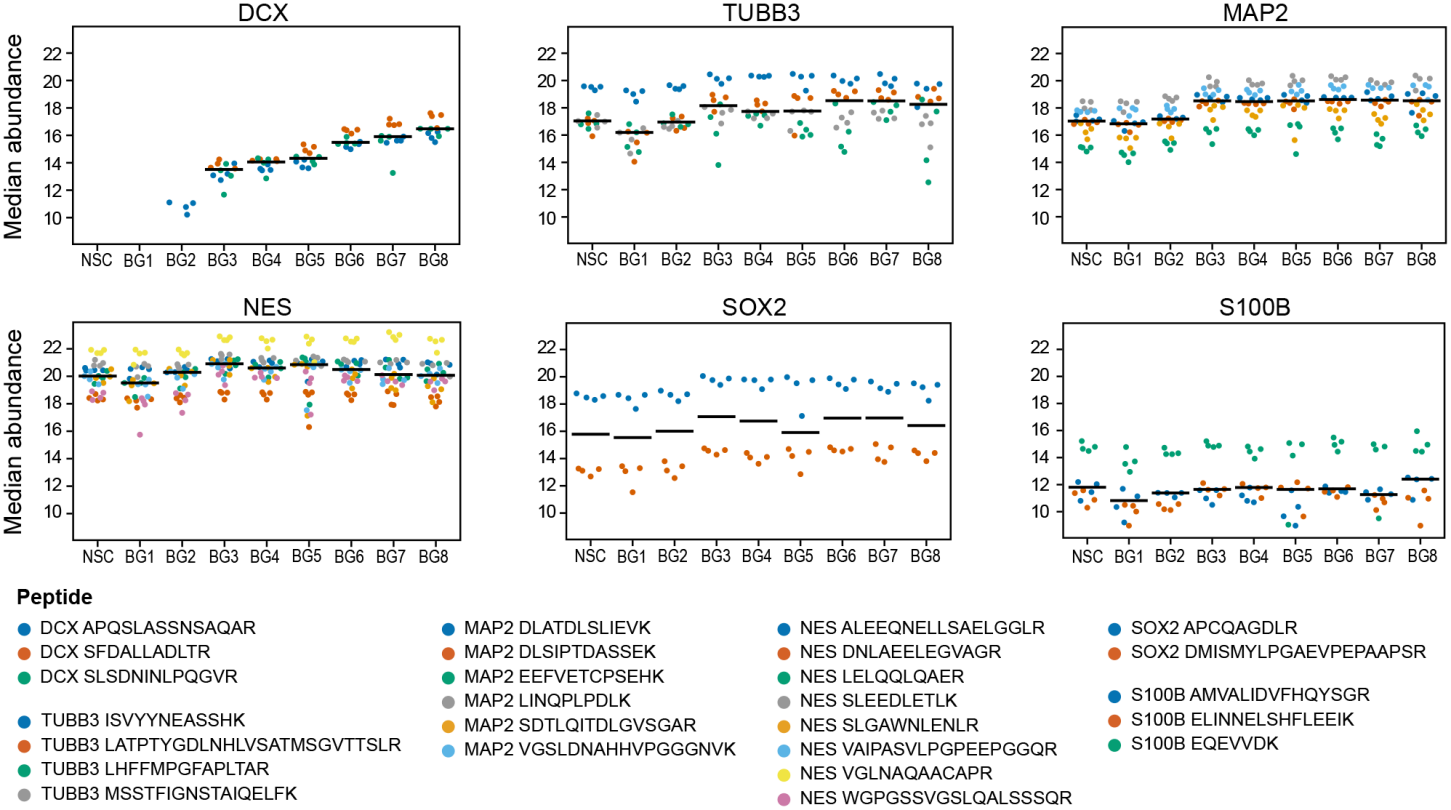
**SRM quantification of 8-day BG differentiation.** Median abundances (black lines) of each protein marker in a given time interval of BG differentiation (1–8 days). Dots of the same colour represent peptide abundances in four biological replicates. This is defined as the median of log2-transformed peak area of all transitions of the same peptide.

Our data indicate that major changes occur in the first week of the BG differentiation (Figs 3 and 4), so we zoomed in, and analysed differentiating NSCs daily for the first 8 days. We found that DCX and TUBB3/MAP2 increased from day 2 and 3, respectively, NES and SOX2 decreased from day 4, and S100B decreased until day 8 (Fig. 5). The expression of neuronal and NSC markers, and the astrocyte marker S100B is regulated at the very early stages of *in vitro* differentiation.

### SRM monitoring of differentiating NSCs, ESCs, and astrocytes

**Fig. 6.**
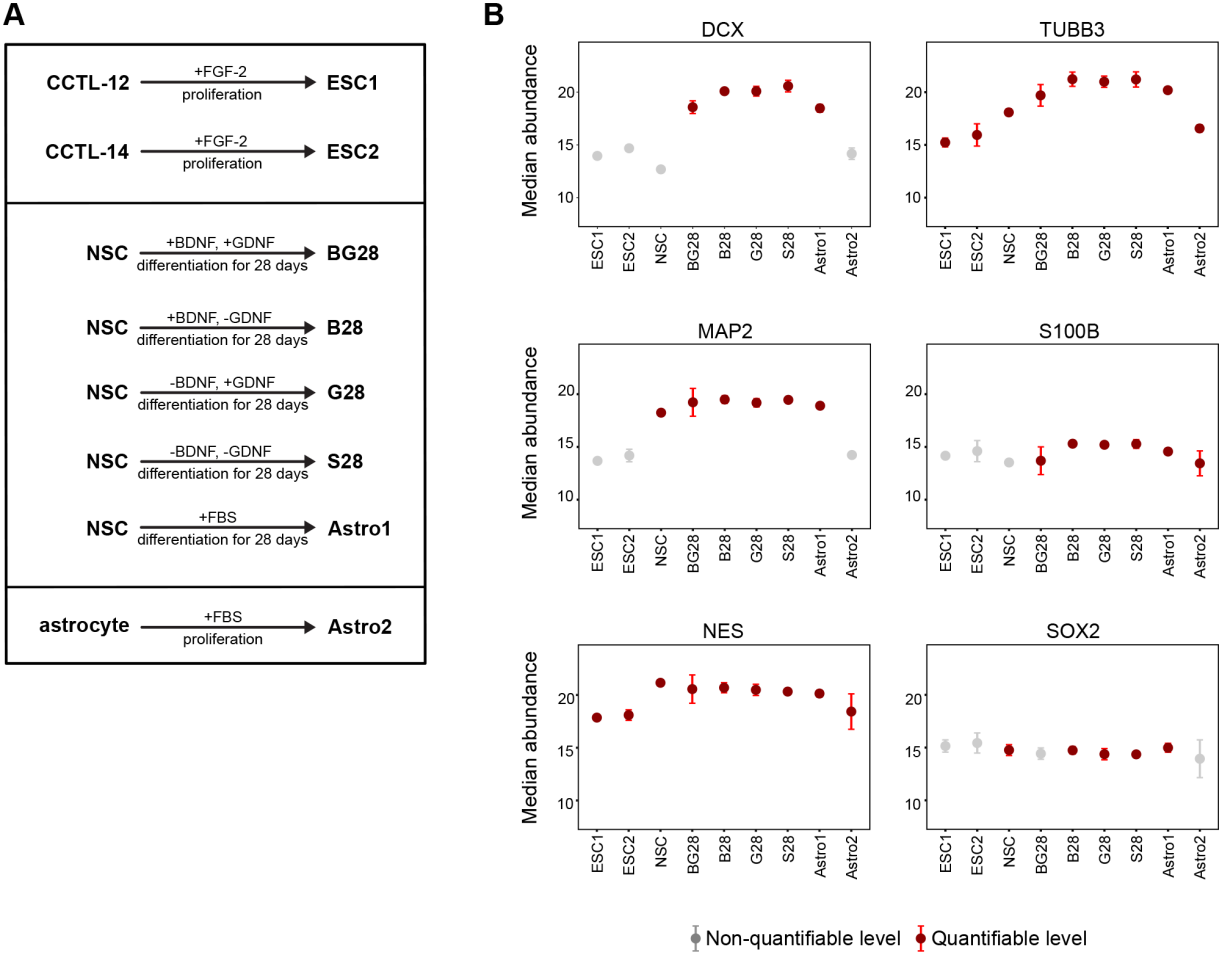
**SRM monitoring of NSC differentiation.** (A) A panel of additional conditions for validation involved two reference ESC lines (ESC1, ESC2), NSCs differentiated with both BDNF and GDNF (BG28), BDNF only (B28), GDNF only (G28), spontaneously differentiated NSCs (S28), astrocyte differentiating NSCs (Astro1), and reference mature astrocytes (Astro2). (B) Condition plots from quadruplicate cultures were generated using MSstats; graphs show median signal and 95% confidence intervals. Red colour means that the protein was quantifiable in this condition (≥2 peptides per protein were detected in ≥3 biological replicates), grey colour means that protein abundances were below quantification levels in particular conditions.

Next, we tested a panel of additional culture conditions. NSCs were directed into neurons using the differentiation medium without EGF and FGF-2 supplemented with different combinations of BDNF and GDNF, and into astrocytes using FBS (Fig. 6A). Our recent data revealed that these neurotrophic factors affected the later stages of differentiation (Červenka et al., 2021), so we employed our SRM assay to depict this effect after 4 weeks of differentiation. As a reference, pluripotent ESCs and mature astrocytes were processed for MS analysis (Fig. 6A).

SOX2, DCX, and MAP2 were quantifiable in the course of neuronal differentiation induced with BDNF and/or GDNF (BG28, B28, G28), in the spontaneously differentiated NSCs (S28), and in the astroglial differentiation induced by FBS (Astro1) (Fig. 6B; Table S6). NES and TUBB3 were detected by SRM at a quantifiable level in all conditions (Fig. 6B), regardless of their expected specificity. OCT4 was quantifiable in the reference ESCs (ESC1, ESC2) and GFAP in the reference astrocytes (Astro2), exclusively (Fig. 6A; Table S6). In agreement with our SRM data (Fig. 6B), the expression of OCT4 pluripotent ESC marker was previously confirmed in the ESC1 and ESC2 cell lines (The International Stem Cell Initiative*, 2007). S100B astrocyte marker was recognized as a suitable protein for quantification in both astrocyte conditions, and in NSCs induced to neuronal differentiation (Fig. 6B).

Our neural cell cultures do not contain detectable amounts of terminally differentiated oligodendrocytes. GALC levels could be quantified by SRM in the pluripotent ESCs and in the astrocyte differentiating NSCs (Table S6), highlighting the validity of this protein as a target for stem cell studies. OLIG1 was identified in differentiating NSCs only as detectable, without possible quantification. This marker was retained in the assay for its prospective use in oligodendrocyte differentiation studies where OLIG1 levels are expected to rise and for its correlation with GALC levels. Protein abundance changes prove the validity of all protein markers, except OLIG1, for their simultaneous quantification by SRM (Table S6).

**Fig. 7.**
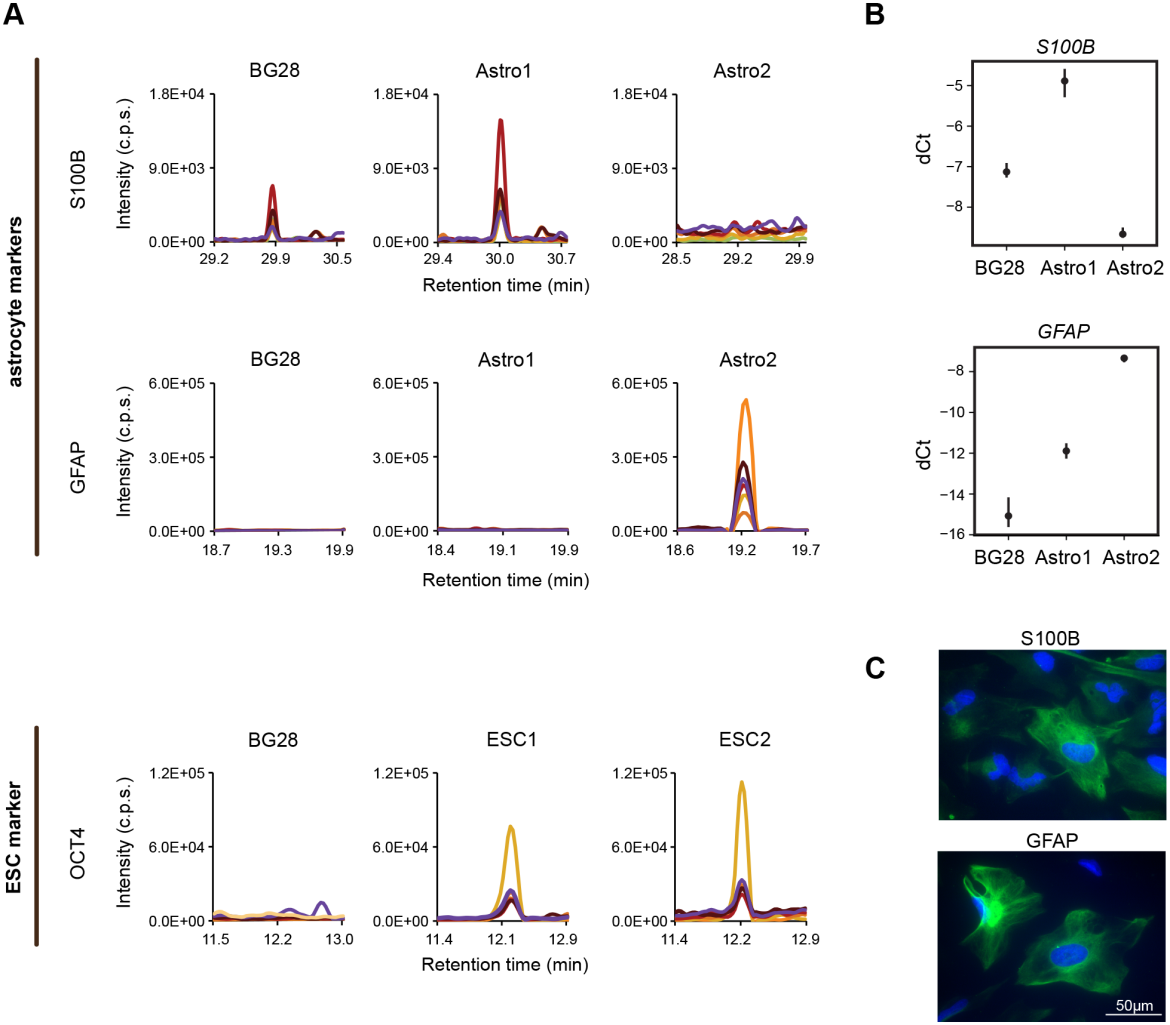
**Astrocyte and ESC markers.** (A) S100B and GFAP astrocyte markers in NSCs directed into astrocytes (Astro1) and in mature astrocytes (Astro2), OCT4 marker of ESCs in reference ESC lines (ESC1, ESC2). Representative endogenous peptides are displayed as a chromatographic trace of peptide elution and detection by SRM. Coloured traces represent the detection of different SRM transitions. (B) *S100B* and *GFAP* mRNAs analysed by RT-qPCR. Individual transcripts were normalized to two housekeeping controls (*GAPDH* and *ATP5F1B*) from three independent experiments. Mean ±95% confidence intervals are shown as black points and vertical lines. (C) Representative IF images of mature astrocytes (Astro2) show GFAP and S100B protein markers in green, cell nuclei counterstained by DAPI in blue. Scale bar: 50 μm. Images of negative controls (no primary antibody) are shown in Table S3 with the table of used antibodies.

The SRM data showed that neuronal markers, and the astrocyte marker S100B were strongly induced, while NSC markers were mostly reduced in all differentiation conditions (Fig. 6B). A weak signal of the astrocyte marker GFAP was detected only in the BG-induced NSCs for one of its unique peptides (Table S6). Different levels of astrocyte markers were identified in the Astro1 cells derived from NSCs and in the Astro2 mature astrocytes (Fig. 7). S100B increased in abundance in the astrocyte differentiating NSCs, but not in the mature astrocytes (Fig. 7A,B). All four peptides of the GFAP marker were detected specifically in the mature astrocytes, but not in the astrocyte differentiating NSCs (Fig. 7A). In mature astrocytes, antibody-based imaging confirmed strongly positive cells for GFAP (Fig. 7C), which was negative in all differentiation conditions of H9-derived NSCs (data not shown). BG cells were positive for S100B (Fig. 2B) without expected morphological changes, compared to mature astrocytes (Fig. 7C). Importantly, proteins marked in our study as quantifiable in the pluripotent ESC1 and ESC2 cells (OCT4, GALC) were also observed in the Astro1 cells exposed to FBS (less defined culture conditions) (Table S6). Based on SRM, we demonstrate that all the differentiation conditions we considered have pleiotropic effects, and simple removal of EGF and FGF-2 is sufficient for triggering neuronal phenotype changes. The astrocyte differentiating H9-derived NSCs manifest rather neuronal than astroglial phenotype.

## DISCUSSION

The animal *in vivo* grafting experiments with human NSCs derived from fetal tissue, ESCs, or iPSCs have accumulated convincing and valuable data to support cell-replacement therapies in neurological disorders and CNS injuries (Cizkova et al., 2007; Hefferan et al., 2012; Jensen et al., 2013; Kelly et al., 2004; Lu et al., 2012, 2014; Svendsen et al., 1997; van Gorp et al., 2013; Yuan et al., 2013). Fetal tissues come with inherent ethical and logistical issues (Barker and de Beaufort, 2013), and it is evident that the source of such a tissue is limited. However, fetal cerebral tissue grafting experiments into human patients with neurodegenerative diseases provided us with invaluable information about feasibility, safety, and experimental procedures. It thus paved the way for the use of proliferating NSC lines generated from a single donor (fetal tissue, embryo, or skin biopsy-reprogrammed cells) that may represent the cells of choice. While the development of new grafting and immunosuppression strategies is ongoing (Bjarkam et al., 2010; Boulis, 2010; Cunningham, 1998; Marsala, 2014; Usvald et al., 2010), it is essential to establish in parallel a reliable and reasonably fast screening protocol that would assess the potential of the selected NSCs as well as their safety.

To address shortcomings of antibody-based screens, targeted MS analysis by SRM can be used not only to accurately verify protein abundance changes emerging from global transcriptome and proteome profiling (Červenka et al., 2021; Donega et al., 2019; Tyleckova et al., 2016; Yocum et al., 2008), but rather to identify markers that would provide a reliable read-out for the differentiation potential of NSCs. We had developed a quantitative high-throughput assay for neuroscience studies, and evaluated its capability to monitor neurogenic potential and maturity of lineage-directed populations of NSCs. A variety of expected responses were detected by SRM, including increased neuronal markers from very early stages of *in vitro* differentiation and decreased NSC multipotency markers in later stages. Persisting expression of NES and SOX2 in differentiated cells might indicate that multipotent NSCs are still present in this population, providing a potential source for ongoing proliferation and differentiation upon transplantation into the host CNS. Alternatively, it may suggest persistent NES and SOX2 expression in non-neuronal populations, e.g. differentiating glia. Either way, it would make NES and SOX2 ideal negative selection markers for pure neuronal populations.

Our data show that DCX, TUBB3, and MAP2 represent more neuro-specific markers compared to NES and SOX2. For this reason, no single marker should be used as definitive proof of a particular cell type. Instead, a quantitative evaluation of several markers in a combinatory assay should be used to identify a protein profile (cell signature) of a selected cell population. Combinatory quantitative assays targeting protein markers may indeed represent a powerful method that would report on the multidifferentiation potential of NSCs, both *in vitro* and *in vivo* (Nagato et al., 2005). Dunkley et al. (2015) introduced a human pluripotent stem cell-derived cellular model of neuronal development. The SRM-based protein profiling applied in this study enabled the identification of time-dependent patterns conserved across multiple cell lines. However, care should be taken to include only reliable and independently verified markers, to avoid measuring uninformative markers.

Ideally, reference pure cell populations would be used as controls for individual markers, but the post-mitotic nature of terminally differentiated neurons and oligodendrocytes makes this impossible for human cells. Immortalized or progenitor human cell lines still depend on *in vitro* differentiation and/or suffer from biased protein patterns (Conti and Cattaneo, 2010; Melo-Braga et al., 2015). FBS had been used in our study to differentiate NSCs into astrocytes, and the OCT4 marker became detectable, indicating the potential of H9-derived NSCs to dedifferentiate and manifest pluripotent traits. The GALC marker did not reach the limit of quantification in the course of BG differentiation, but appeared at a quantifiable level in pluripotent ESCs and in the FBS-induced NSCs.

S100B levels varied considerably in H9-derived NSCs exposed to various stimuli. Although S100B is a broadly accepted marker of astroglial cells, its dynamic expression was reported in NSCs in developing rat brain (Patro et al., 2015), and in human NSC lines (Lam et al., 2019). S100B expression in rat neural progenitor cells correlated with their proliferative potential. When the progenitor cells had stopped dividing, S100B was downregulated, and its expression was restored in mature astrocytes, together with an astrocyte marker GFAP (Patro et al., 2015). GFAP was readily detected in our mature astrocytes, and was at detection, but not quantification limit in BG neuronal differentiation cells, demonstrating the presence of sporadic astrocytes in this population. As we reported recently, NSCs derived from human ESCs showed no detectable GFAP signal during 3–6 weeks of the FBS-induced *in vitro* differentiation. However, 2–6 months after *in vivo* grafting into immunosuppressed rats and minipigs, a high number of GFAP positive human astrocytes is clearly detectable (Bohaciakova et al., 2019). These findings resemble *in vivo* embryonal development of the human cerebral cortex, where no expression of GFAP was detectable at week 11, whereas S100B was expressed (Vinci et al., 2016). Our results suggest that classical protocols are not optimal for *in vitro* differentiation of mature astrocytes from the ESC-derived NSCs.

Here we show that the SRM-based quantification of suitable proteins/peptides is a powerful tool to report on the presence of pluripotent, multipotent, and committed neuronal and glial cells. SRM allows fast and reproducible detection of a predefined set of proteins, spanning a broad range of abundances (Lange et al., 2008). Some of the potential specific markers were not accessible by SRM due to the lack of tryptic peptides specific for a single protein with respect to the human proteome (NANOG). For other targets, specific SRM assays could be developed using synthetic heavy-labelled peptides, but were below the limit of detection in our cultures (PAX6, MKI67, VEGF-A, and CXCL1). These proteins can be substituted by other markers, such as minichromosome maintenance complex components as additional markers of proliferation, or other relevant proteins expected from the literature to report on neural (stem) cell populations and their derivatives (Zizkova et al., 2015).

The quantitative SRM assay presented here can be applied to an unlimited number of human NSC lines at high throughput and reproducibility, and using ∼300,000 cells to be able to perform this high-accuracy quantitative measurement repeatedly. Since the *in vitro* differentiation takes long periods of time, we suggest running the assay before commencing large-scale experiments to ensure high reproducibility. On top of that, relatively low cell numbers required for a successful SRM measurement brings the possibility of using identical samples for multiple high-throughput screens, including bulk or single-cell deep RNA sequencing, thus offering unique gene/protein expression cross-validation.

We propose the application of the developed neural cell SRM assay as quality control for optimizing culture conditions during NSCs propagation and differentiation. The multiplexing capacity enables to include broad spectra of targets (∼150 proteins) that could be selected from relevant molecular pathways (e.g. cell cycle, apoptosis, stress response, etc.) and measured together with the current panel of markers within the 30-min MS method (Soste et al., 2014). However, novel candidate markers need to be screened for their biological relevance and MS detectability with respect to the number of targeted peptides and their suitability for quantification. Fluorescence activated cell sorting (FACS) analysis of proliferating NSCs could increase the throughput in the SRM assays validation step, which could be further combined with a sorting strategy coupled to SRM. This would make it possible to distinguish between maturity and purity of neural populations generated from NSCs, adding another level of information.

### Conclusions

In summary, we developed a novel SRM-based assay that could be easily employed to assess the neurogenic/gliogenic potential of NSCs during the propagation phase. The assay can be further exploited in *in vitro* experiments which could lead to improved or even novel differentiation protocols. The sensitivity and speed could eventually allow for testing of banked NSCs to test their differentiation potential upon long-term storage. Moreover, the SRM assay can be simply adapted to the analysis of additional cell types and experimental approaches.

## MATERIALS AND METHODS

### Neural stem cells differentiation

Unless otherwise stated, cell culture reagents were obtained from Life Technologies (Thermo Fisher Scientific Inc., Waltham, MA, USA). Cells were maintained at 37°C in 5% CO_2_ in a humidified atmosphere.

Gibco Human Neural Stem Cells (H9-derived) generated from the NIH approved human ESCs (WA09; 46, XX) had been obtained from Life Technologies (catalogue number 510088, lot number 1402001, Thermo Fisher Scientific Inc.) and cultured as described previously (Červenka et al., 2021) with modifications. Briefly, the H9-derived NSCs (condition NSC) were grown on 20 μg/ml poly-L-ornithine and 5 μg/ml laminin-coated plates (both from Sigma-Aldrich, St. Louis, MO, USA) in the NSC proliferation medium containing KnockOut Dulbecco's modified Eagle's medium (DMEM)/F-12, 2 mM GlutaMAX, 1% penicillin-streptomycin, 2% StemPro Neural Supplement, 20 ng/ml human recombinant FGF-2, and 20 ng/ml human recombinant EGF. The NSC proliferation medium was changed every other day, and cells were passaged every 5–7 days using 0.05% trypsin/ethylenediaminetetraacetic acid (EDTA). Once the NSC culture had been established, low-passage cells were directed toward a specific lineage using an appropriate differentiation medium.

For directed differentiation into neurons, the NSC proliferation medium was switched to neuronal differentiation medium by exchanging FGF-2 and EGF for human recombinant BDNF and human recombinant GDNF (10 ng/ml of each; both from PeproTech, Rocky Hill, NJ, USA). Half of the differentiation medium was changed every other day, directing the NSC differentiation into neurons upon the treatment with BDNF and GDNF for 7, 14, 21, and 28 days (conditions BG7, BG14, BG21, BG28).

In the zooming experiment, NSCs were supplemented with BDNF and GDNF for 1–8 days (conditions BG1-8).

To evaluate the effect of BDNF and GDNF, these factors were applied exclusively for 28 days (conditions B28, G28). For spontaneous neuronal differentiation, neither BDNF nor GDNF was used, and NSCs differentiated by removing FGF-2 and EGF (condition S28).

For differentiation into astrocytes, NSCs were grown on Geltrex-coated plates, and the NSC proliferation medium was switched to astrocyte differentiation medium containing KnockOut DMEM/F-12, 1% N-2 Supplement, 2 mM GlutaMAX, 1% FBS, 1% penicillin-streptomycin. The astrocyte differentiation medium was changed every 3–4 days, directing the NSC differentiation into astrocytes for 28 days (condition Astro1).

### Reference cell populations

Gibco Human Astrocytes generated from brain progenitor-derived astrocytes were obtained from Life Technologies (part number K1884, lot number 1640797, Thermo Fisher Scientific Inc.). Astrocytes (condition Astro2) were cultured according to the manufacturer's instructions on Geltrex-coated plates in the astrocyte proliferation medium containing Gibco Astrocyte Medium, 1% N-2 Supplement, 1% penicillin-streptomycin, and 10% FBS. The astrocyte proliferation medium was changed every other day, and cells were passaged every 3–4 days using 0.05% trypsin/EDTA.

Human ESC lines CCTL-12 [46, XX, del(18); condition ESC1] and CCTL-14 (46, XX; condition ESC2) (The International Stem Cell Initiative*, 2007) were grown on gelatin-coated plates in the presence of mitotically inactivated primary mouse embryonic fibroblasts (derived from 12.5-day-old mouse embryos, strain CF1; density 24,000 cells/cm^2^). DMEM/F-12 was supplemented with 15% knockout serum replacement, 2 mM L-Glutamine, 1× minimum essential medium non-essential amino acids, 0.5% penicillin-streptomycin, 100 µmol/lβ-2 mercaptoethanol (Sigma-Aldrich), and 4 ng/ml FGF-2 (PeproTech). The embryonic culture medium was changed every day, and cells were manually passaged every 5–7 days. For sample preparation, ESC colonies were manually detached from the cell culture dish to avoid contamination with mouse embryonic fibroblasts.

### Immunocytochemistry

Selected markers were monitored in BG-differentiating NSCs and in astrocyte conditions by IF imaging. Cells were seeded on Nunc Lab-Tek chambered slides. Cells cultured as described above were washed with pre-heated phosphate-buffered saline (PBS), fixed with 4% paraformaldehyde in PBS for 15 min, and washed three times with PBS. The cells were then permeabilized and blocked with 0.1% Triton X-100, 5% goat serum, and 1% bovine serum albumin in PBS for 45 min. For the cell surface marker GALC, Triton X-100 was omitted. Antibodies (Table S3) were diluted in 5% goat serum in PBS and incubated with the cells overnight at 4°C. After three washing steps with PBS, antibodies were detected using fluorescently-labelled secondary antibodies (goat anti-mouse or goat anti-rabbit; Alexa Fluor 488; both from Thermo Fisher Scientific Inc.) diluted to 1:500 in 5% goat serum in PBS for 60 min in the dark. After three washing steps with PBS, DNA was stained with DAPI. In the negative controls, primary antibodies were omitted (Table S3). Fluorescent images were captured using an inverted fluorescent microscope in 16-bit depth (DMI6000 B; Leica Microsystems, Wetzlar, Germany) and assembled in ImageJ software (v1.49k, National Institutes of Health, Bethesda, MA, USA) (Schneider et al., 2012).

### Quantitative reverse transcription PCR

Gene expression analyses of selected markers were performed as described previously (Červenka et al., 2021). Briefly, total RNA was isolated from BG-differentiating NSCs and astrocyte conditions by RNeasy Plus Mini Kit (Qiagen) with QIAshredder (Qiagen), and converted into cDNA with QuantiTect Reverse Transcription Kit (Qiagen) according to the manufacturer's instructions. The reaction mix for one quantitative PCR contained 5× HOT FIREPol EvaGreen qPCR Mix Plus (Solis BioDyne), 125 nM of each primer (Table S4), 25 ng of cDNA template, and PCR water. Following settings were used on CFX96 Touch Real-Time detection system (Bio-Rad): 12 min at 95°C for enzyme activation, then 15 s at 95°C for denaturation with 40 cycles of 30 s at 57°C for annealing, and 30 s at 72°C for an extension. Cycle threshold (Ct) values were normalized to the average of two housekeeping genes Glyceraldehyde-3-phosphate dehydrogenase (*GAPDH*) and ATP synthase subunit beta, mitochondrial (*ATP5F1B*).

### Sample preparation for MS analysis

Cell samples in four bioreplicates for each condition were washed with PBS and resuspended in a buffer containing 8 M urea (Sigma-Aldrich), 50 mM ammonium bicarbonate (NH_4_HCO_3_, Sigma-Aldrich), and 5 mM EDTA (Carl Roth GmbH, Karlsruhe, Germany). The cells were disrupted by vortexing (ten consecutive rounds of 1.2 min) and by sonicating on ice (15 min). The samples were centrifuged at 20,000 ***g*** for 15 min (4°C) to remove any remaining debris, and protein concentrations were determined (Pierce 660 nm Protein Assay, Thermo Fisher Scientific Inc.). The protein extracts were then supplemented with ProteaseMAX Surfactant (Promega, Madison, WI, USA) to a final concentration of 0.1%. After vortexing and sonicating as described above, proteins were reduced with 10 mM tris(2-carboxyethyl)phosphine for 30 min at 32°C and alkylated with 40 mM iodoacetamide for 45 min at 25°C, in the dark. Samples were diluted with freshly prepared 0.1 M NH_4_HCO_3_ and 0.01% ProteaseMAX to a final concentration of 1 M urea, and incubated at 37°C with sequencing-grade Lysyl Endopeptidase (Wako Chemicals GmbH, Neuss, Germany) and sequencing-grade porcine trypsin (Promega) proteases for 4 h and 14 h, respectively, both in an enzyme/substrate ratio of 1/100 (w/w). The digestion was stopped by acidification with formic acid (FA) to a final pH <3. The peptide mixtures were loaded onto C18 spin columns (The Nest Group Inc., Southborough, MA, USA) to desalt according to the manufacturer's instructions. Peptides were eluted with 80% acetonitrile. Peptide samples were desiccated on a vacuum centrifuge and re-solubilized in 0.1% FA for LC-MS analysis. Samples were processed in parallel with their respective controls (NSCs).

### SRM assays development

Proteotypic peptides matching 14 frequently used protein markers of the NSC differentiation (Table S1) were retrieved from publicly available resources of targeted proteomics assays (SRM Atlas, http://www.srmatlas.org/). Development and validation of SRM assays to measure protein abundances were performed as previously described (Soste et al., 2014) using heavy-labelled unpurified synthetic peptides (Thermo Scientific Biopolymers, Thermo Fisher Scientific Inc.). These were mixed and monitored by LC-SRM using a 5500 QTrap triple-quadrupole/ion-trap mass spectrometer (Sciex, Framingham, MA, USA) equipped with a nano-electrospray ion source (Sciex). On-line chromatographic separation of the peptides was achieved with an Eksigent 425 nanoLC system (Eksigent/Sciex) equipped with a 20-cm fused-silica column with a 75-μm inner diameter (New Objective, Woburn, MA, USA), packed in-house with ProntoSIL C18 AQ 3 μm beads (Bischoff Analysentechnik GmbH, Leonberg, Germany). The peptide mixtures were loaded and separated with a linear gradient from 5% to 35% acetonitrile over 30 min at a flow rate set to 350 nl/min. The instrument was operated as described in Soste et al. (2014). SRM analysis was conducted with Q1 and Q3 operated at unit resolution (0.7 m/z half-maximum peak width) with a dwell time of 10 ms and a cycle time ∼3.5 s. For each peptide, doubly and triply charged precursor ions, and the 20 most probable singly or doubly charged fragment ions from the b- and y-ion series were selected using Skyline (v3.1.1.7490, release date 20 May 2015, MacCoss Lab Software, University of Washington, Seattle, WA, USA) (MacLean et al., 2010) and measured by SRM. The indexed retention time peptides (Biognosys AG, Zürich, Switzerland) were annotated and used to schedule the acquisition of selected SRM traces within retention-time (RT) windows. Synthesized peptides containing a heavy-isotope label were then spiked into cell samples, and corresponding heavy and light transitions were targeted to monitor the co-elution of endogenous (i.e. light) peptides and the spiked-in (heavy) surrogates in different conditions. The raw data can be accessed at http://www.peptideatlas.org/PASS/PASS00872. Data were analysed with Skyline, and ten validated markers represented by ≥2 proteotypic peptides per protein and the ≥4 most suitable transitions (precursor and fragment ion pairs) per peptide were experimentally selected for quantification experiments.

### Protein quantification

To track the system performance, commercial predigested Beta-Galactosidase (Sciex) was diluted with indexed retention time peptides (Biognosys AG) and Glu-1-Fibrinopeptide B (Sciex) to a working solution of 20 fmol/μl, and monitored prior to analysis. In time-scheduled SRM experiments, protein markers were targeted in all bioreplicates of each condition (total 258 transitions, 4-min RT window, 1.7-s cycle time, 1 μg peptides, 30-min gradient) (Table S5). Corresponding conditions and controls (NSCs) were analysed with the same RT window using the instrument settings described above. The raw data can be accessed at http://www.peptideatlas.org/PASS/PASS00873. SRM peaks were manually inspected using Skyline by checking for co-elution, peak shape similarity, a match in relative intensities of fragment ions and retention times compared to the assay development phase. Only SRM peaks detected with a signal-to-noise ratio of >3 for at least the top transition were considered for quantification. Raw SRM data (peak areas) were exported from Skyline and, for transitions below the transition specific background level, the peak areas were assigned to one-third of the background level. Protein significance analysis was performed using an open-source statistical environment R (R Core Team, 2020) (version 4.0.2) with package MSstats (version 3.20.1), which combines the quantitative measurements for peptides, charge states, and transitions, and detects proteins that change in abundance between conditions while controlling the false discovery rate (Chang et al., 2012; Choi et al., 2014). The peptide and protein abundances were calculated from log2-transformed peak areas of individual transitions. A linear mixed-effects model was used for the relative quantification of a given condition with respect to its control (NSCs). Significant abundance changes were reported as log2 fold-changes with standard error, T value, degrees of freedom, and *P*-value adjusted for multiple comparisons (Table S6). A false discovery rate-adjusted *P*-value cut-off of 0.05 was used. The R environment (R Core Team, 2020) was used to generate a variety of different plots.

## Supplementary Material

Supplementary information
