# Supplementary material for: Targeted mass spectrometry for monitoring of neural differentiation
Source: Biol Open. 2021 Aug 6;10(8):bio058727. doi: 10.1242/bio.058727 (PMC8353267; doi:10.1242/bio.058727)
Supplement: Supplementary information [file biolopen-10-058727-s1.pdf]

Table S1. Markers routinely used to probe neuronal differentiation.

Click here to download Table S1

Table S2. Targeted peptides.

Click here to download Table S2

Table S3. Antibodies used in the present study.

Click here to download Table S3

Table S4. Primers used for quantitative PCR analyses.

Click here to download Table S4

Table S5. Complete set of SRM assays.

Click here to download Table S5

Table S6. SRM quantification data across NSC differentiation experiments.

Click here to download Table S6

Table S7. SRM to qPCR data correlation.

Click here to download Table S7

Biology Open • Supplementary information
